# Extended Perfusion Parameter Estimation from Hyperspectral Imaging Data for Bedside Diagnostic in Medicine

**DOI:** 10.3390/molecules24224164

**Published:** 2019-11-17

**Authors:** Jörg Marotz, Axel Kulcke, Frank Siemers, Diogo Cruz, Ahmed Aljowder, Dominik Promny, Georg Daeschlein, Thomas Wild

**Affiliations:** 1 Klinik für Plastische und Handchirurgie und Brandverletztenzentrum, BG-Klinikum Bergmannstrost, D-06002 Halle (Saale), Germany; frank.siemers@bergmannstrost.de; 2 Institute of Applied Bioscience and Process Management, University of Applied Science Anhalt, D-06366 Köthen (Anhalt), Germany; thomas.wild@woundconsulting.com; 3 Diaspective Vision GmbH, D-18233 Am Salzhaff, Germany; axel.kulcke@diaspective-vision.com; 4 Clinic of Plastic, Hand and Aesthetic Surgery, Medical Center Dessau, University of Applied Science Anhalt, D-06847 Dessau, Germany; diogo.cruz@klinikum-dessau.de; 5 Clinic of Dermatology, Immunology and Allergology, Medical Center Dessau, Medical University Brandenburg “Theodor Fontane“ Medical Center Dessau, D-06847 Dessau, Germany; ahmedaljowder@hotmail.com; 6 Klinik für Plastische, Wiederherstellende und Handchirurgie, Zentrum für Schwerbrandverletzte, Klinikum Nürnberg, D-90471 Nürnberg, Germany; dpromny@gmail.com; 7 Klinik und Poliklinik für Hautkrankheiten, Universitätsmedizin Greifswald, D-17475 Greifswald, Germany; Georg.Daeschlein@med.uni-greifswald.de

**Keywords:** hyperspectral image processing, perfusion measurements, clinical classifications

## Abstract

**Background:** Hyperspectral Imaging (HSI) has a strong potential to be established as a new contact-free measuring method in medicine. Hyperspectral cameras and data processing have to fulfill requirements concerning practicability and validity to be integrated in clinical routine processes. **Methods:** Calculating physiological parameters which are of significant clinical value from recorded remission spectra is a complex challenge. We present a data processing method for HSI remission spectra based on a five-layer model of perfused tissue that generates perfusion parameters for every layer and presents them as depth profiles. The modeling of the radiation transport and the solution of the inverse problem are based on familiar approximations, but use partially heuristic methods for efficiency and to fulfill practical clinical requirements. **Results:** The parameter determination process is consistent, as the measured spectrum is practically completely reproducible by the modeling sequence; in other words, the whole spectral information is transformed into model parameters which are easily accessible for physiological interpretation. The method is flexible enough to be applicable on a wide spectrum of skin and wounds. Examples of advanced procedures utilizing extended perfusion representation in clinical application areas (flap control, burn diagnosis) are presented.

## 1. Introduction

Hyperspectral Imaging (HSI, imaging remission spectroscopy, or diffuse reflectance spectroscopy) as a non-contact, stressless imaging measuring method is currently an intensively developing area for diverse medical applications [1,2]. Despite the limited penetration depth in biological tissue in the visible (VIS) and near infrared (NIR) spectral range, the effect of the specific scattering and absorption by tissue components in this “diagnostic window” makes it possible to retrieve information of significant clinical value [3,4,5,6,7,8,9,10,11].

In perfused tissue like human skin, the remission spectra are mainly influenced by hemoglobin (oxygenated and reduced) absorption. Additional components such as the collagen matrix, melanin, fat, and water contribute by specific scattering and absorption processes. The main focus is the estimation of the perfusion-related parameters of skin or similar tissue systems. Those parameters make it possible to evaluate local (for instance wounds) and regional (for instance PAD, diabetic foot) perfusion quality, and often also systemic attributes of the blood supply and oxygen usage [12,13,14]. Normally in clinical practice, no other methods are available to gain such information in a quick and simplified manner.

For the estimation of perfusion parameters (volume fraction blood, oxygen saturation hemoglobin), sample one- or two-layer models of the tissue with infinite depth and homogeneous distribution of the components are frequently used. Additionally, from the NIR-part of the spectrum, the volume fraction of water can be estimated [15]. The drawback from this is the substantial simplification of the normally complex layered structure of skin and similar tissue systems. The penetration depth of the light, and therefore the measuring volume, depends on the spectral range (VIS: <1 mm, NIR: 4–6 mm in skin) caused by the specific spectral scattering and absorption in the tissue layers. In real layered tissue systems, different layers contribute to a remission signal, depending on the wavelength. Thus, the remission spectrum is a heterogeneous spectrum in relation to the measured volume. The perfusion parameters estimated by these models are values averaged over different layers with unknown weights. With these models and estimated model parameters, normally, the measured spectrum cannot be reproduced, indicating a loss of information.

Nevertheless, even those parameters have been proven to represent a considerable information profit, and to provide additional value for diverse clinical application areas [9,10,11,13,15,16,17].

Recently, compact and cost-efficient hyperspectral cameras for routine clinical practice have been made available. The practicability for clinical use is accomplished by means of a simple measuring process with laminar illumination, direct imaging by an integrated scanning process generating a “3D-data cube“(x-y-λ), fast acquisition of a large area (i.e., approximately 5 seconds for 20 × 30 cm), and no special measuring conditions (beside the avoidance of external light on the measuring area) [14].

In order to establish such easy-to-use cameras, and therefore, hyperspectral imaging technology in a clinical environment, the potentially high information content of the measurement has to be exploited and presented to the clinical user in an informative manner.

Only clinical applications concerning the skin are considered. Mainly quantitative information about the perfusion situation should be generated. In this context, the estimation of the delivered arterial blood quantity and oxygen saturation, as well as the oxygen consumption in the capillary system of the measured area, are of special interest. The imaging measurement additionally allows for the analysis of regional distributions of the perfusion quality and the identification of regional perfusion distortions.

Besides the assessment of the intact skin, perfusion analyses of wounds generally are of special interest, because their quality is an essential factor in wound healing processes. Therefore, the parameter estimation method should be applicable to a wide variety of perfused tissue systems.

Although analytical solutions of the light transport equations in the diffusion approximation in tissue systems are available [18,19], the measuring geometries often do not correspond to the use of a HSI-camera in a clinical environment [20,21]. Simple models like two- or three-layer systems cannot adequately represent the complexity and variability of real skin systems and generate parameters of limited comparability. Solution procedures of the inverse problem (calculation of the model parameters from the measured spectrum) for more realistic multilayer models are still computationally expensive [20]. Also, solution procedures based on artificial neural networks (for instance, seven-layer models require the reduction of the number of parameters) to be efficient, and therefore, do not always adequately describe physiological conditions [22].

We present an evaluation procedure for the remission spectra of skin and wounds, which transforms all the information about the spectrum consistently into model parameters, which are then easily accessible to physiological interpretation. However, we do not claim to generate an exact solution to the problem of the realistic modeling of actual tissue systems.

Consistent transformation means that the measured spectrum should be completely reproduced by the model and the determined model parameters. The consistently-determined model parameters should be used as a more interpretable basis for further clinical estimations, as, for instance, in classification procedures.

The objective is to exploit the information of HSI-measurements in consideration of clinical demands and to create data processing of high practicability.

## 2. Results

### 2.1. D-Physiological Perfusion Imaging

The model-based processing described in Section 4 provides “depth profiles” for perfusion parameters vHb and xHbO_2_ (with six values in each case), one value for vH_2_O, and one for vFat, calculated from Λ_5_ and Λ_6_. Furthermore the depth profiles of the intrinsic structure parameters (s0, s1), as well as the relative “layer thickness“ d_i_ ($d_{i}=D_{i}-D_{i-1},$ relative to $D_{1}^{0}$), are available.

The profiles are presented independently from the layer thickness as a series of parameter values (bars) (see Figure 6). The values of vHb are scaled according to the layer thicknesses determined by the procedure. This form of presentation has been proved to be the most informative in practice.

From the perfusion profiles (for every image pixel), four survey images are generated, depicting vHb and xHbO_2_ for the upper layers 1 and 2 (vHb_1, xHbO_2__1) and for the deeper layers 5 and 6 (vHb_2, xHbO_2__2). The values are color-coded in blue (low), via green (normal) to red (high).

To evaluate the physiological interpretation and validity of the model parameters, the spectra of normal perfused skin (healthy volunteers) and from patients in different clinical areas are recorded and the depth profiles analyzed and proved in terms of their physiological plausibility.

#### 2.1.1. Example: Occlusion Test

**Figure 1:**
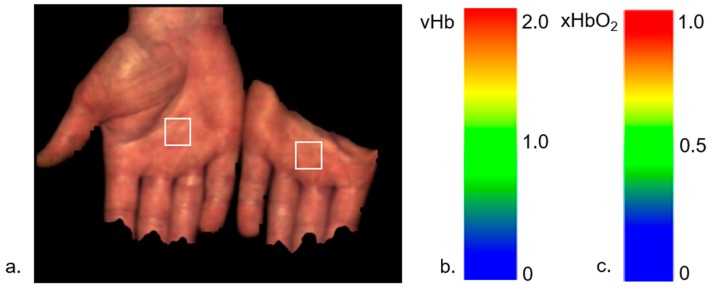
(**a**) Measurements of the hands in an occlusion test; right: hand of the occluded arm, left: reference hand; the white quadrates indicate the tested areas from which the profiles in Figure 3 were determined; (**b**) color scale vHb [0...2], c: color scale xHbO_2_ [0…1].

**Figure 2:**
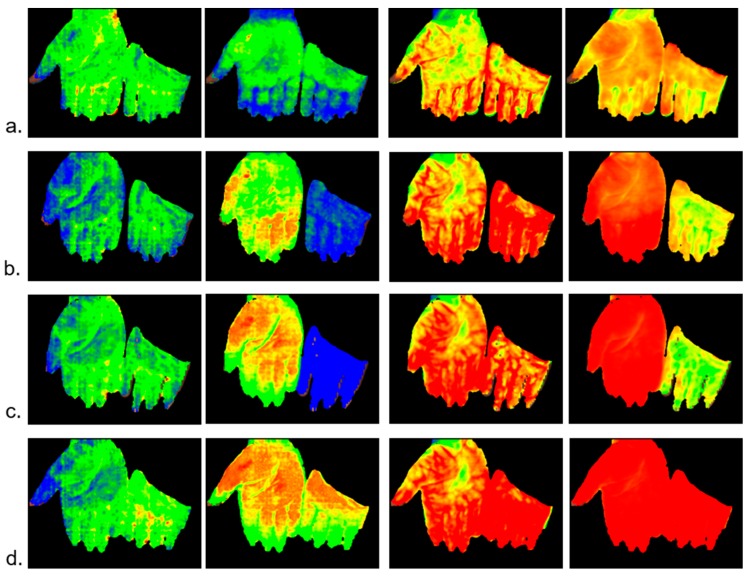
Survey images; color-coded parameters from left to right: vHb_1, xHbO_2__1 (superficial), vHb_2, xHbO_2__2 (deep); (**a**) normal perfusion, (**b**) venous occlusion, (**c**) arterial occlusion, (**d**) reperfusion after arterial occlusion. Color scales for vHb and xHbO_2_, as depicted in Figure 1.

**Figure 3:**
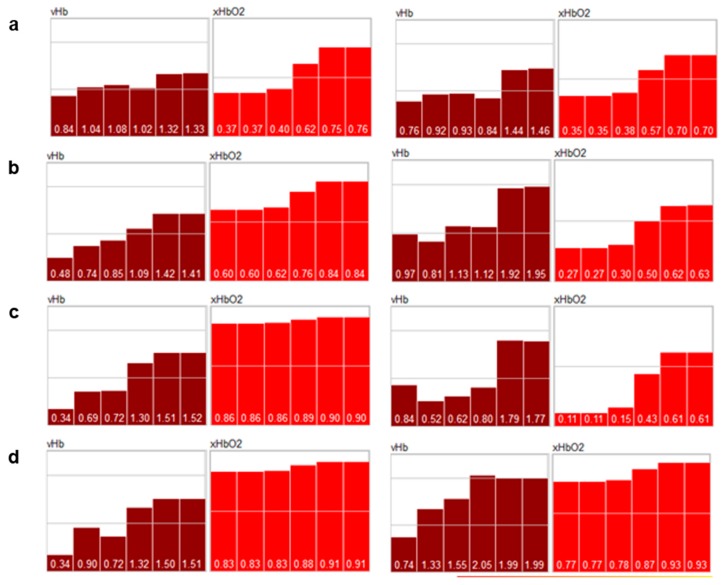
Perfusion profiles averaged over the test areas in Figure 1; the profiles depict the vHb- and HbO_2_-values for 6 depth layers (layer 3 in Figure 8 has been split into 2 layers 3.1 and 3.2) from left to right; the thickness of the layers are not depicted; vHb are index values [0…2.5], xHbO_2_: [0...1]; left column: reference hand; right column: test hand; (**a**) normal perfusion, (**b**) venous occlusion, (**c**) arterial occlusion, (**d**) reperfusion after arterial occlusion.

As a first example, the data from an occlusion test with healthy volunteers are presented. The left arm has been occluded (venous and arterial) and the hands were measured with a HSI camera with the right hand as a reference (Figure 1). The occlusion test contains four phases: normal perfusion, venous occlusion, arterial occlusion, and reperfusion after arterial occlusion. The survey images are shown in Figure 2, and the depth profiles from the test areas in Figure 3. 

The survey images (Figure 2) clearly show the reaction of vHb and xHbO_2_ in the different phases.

The depth profiles of the parameters can be plausibly explained as follows:

“Normal“ perfusion (a): the profiles from the reference and the test hand are similar; vHb shows the normal distribution over the layers 1–6; xHbO_2_ in the superficial layers (1 and 2) is approx. 0.36 due to oxygen consumption in the capillary system; xHbO_2_ in the deep layers (5 and 6) are a mixture of arterial (approx. 98%) and venous blood (0.36) from the capillary system; in the reticular system, the volume fraction of both arterial and venous blood are principally equal (in stationary states), so that xHbO_2_ is the mean value of venous and arterial xHbO_2_;

Venous occlusion (b): vHb increases in all layers, but mainly in 5 and 6, because blood cannot flow off; xHbO_2_ decreases due to consumption and because the arterial supply is also hindered by venous occlusion, but there is still an arterial pressure in the capillary system;

Arterial occlusion (c): no blood flow; the available blood is gathered in the deeper vessels (layers 5 and 6); vHb in layers 1 and 2 is lower than for venous occlusion because of a lower arterial pressure; xHbO_2_ strongly decreases due to consumption;

Reperfusion (d): expansion of all vessels, high blood flow; due to the high flow xHbO_2_ increases in the capillary system because (stationary state) xHbO_2_ in the superficial layers depends of the blood flow.

It is interesting to note the systemic reaction on the occlusion observable in the reference hand; the systemic blood flow increases in the deeper vessel system, while the superficial vHb (layers 1 and 2) decreases in the reference hand; due to the high flow, xHbO_2_ increases.

After the reperfusion phase, the perfusion returns to normal values.

#### 2.1.2. Example: Flap Transplant for Wound Coverage

In the following example, the perfusion evolution of a skin graft over twelve days is shown (measurement each second day).

The depth profiles are available for every point on the flap, and can be used to analyze the perfusion quality and distribution over the flap in detail over time. With close-meshed measurements, over time, developing perfusion problems can be detected and evaluated very early.

**Figure 4:**
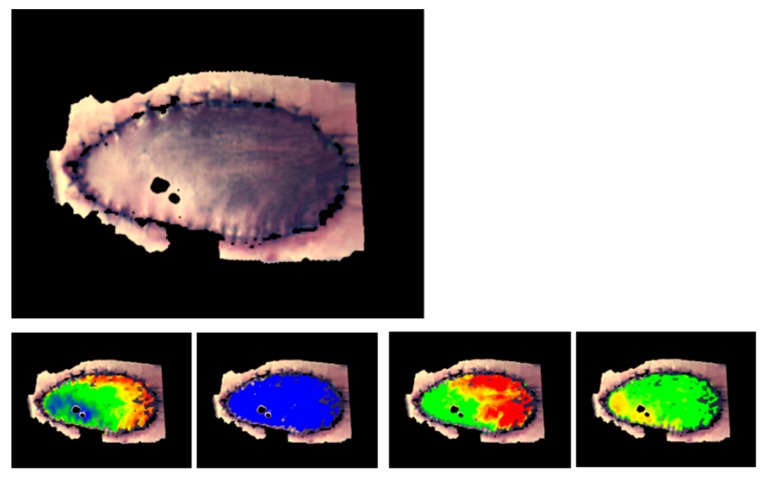
Transplant at day 1; survey images vHb_1, xHbO_2__1, vHb_2, xHbO_2__2; the arterial influx is on the right side of the flap; color scales for vHb and xHbO_2_, as depicted in Figure 1.

**Figure 5:**
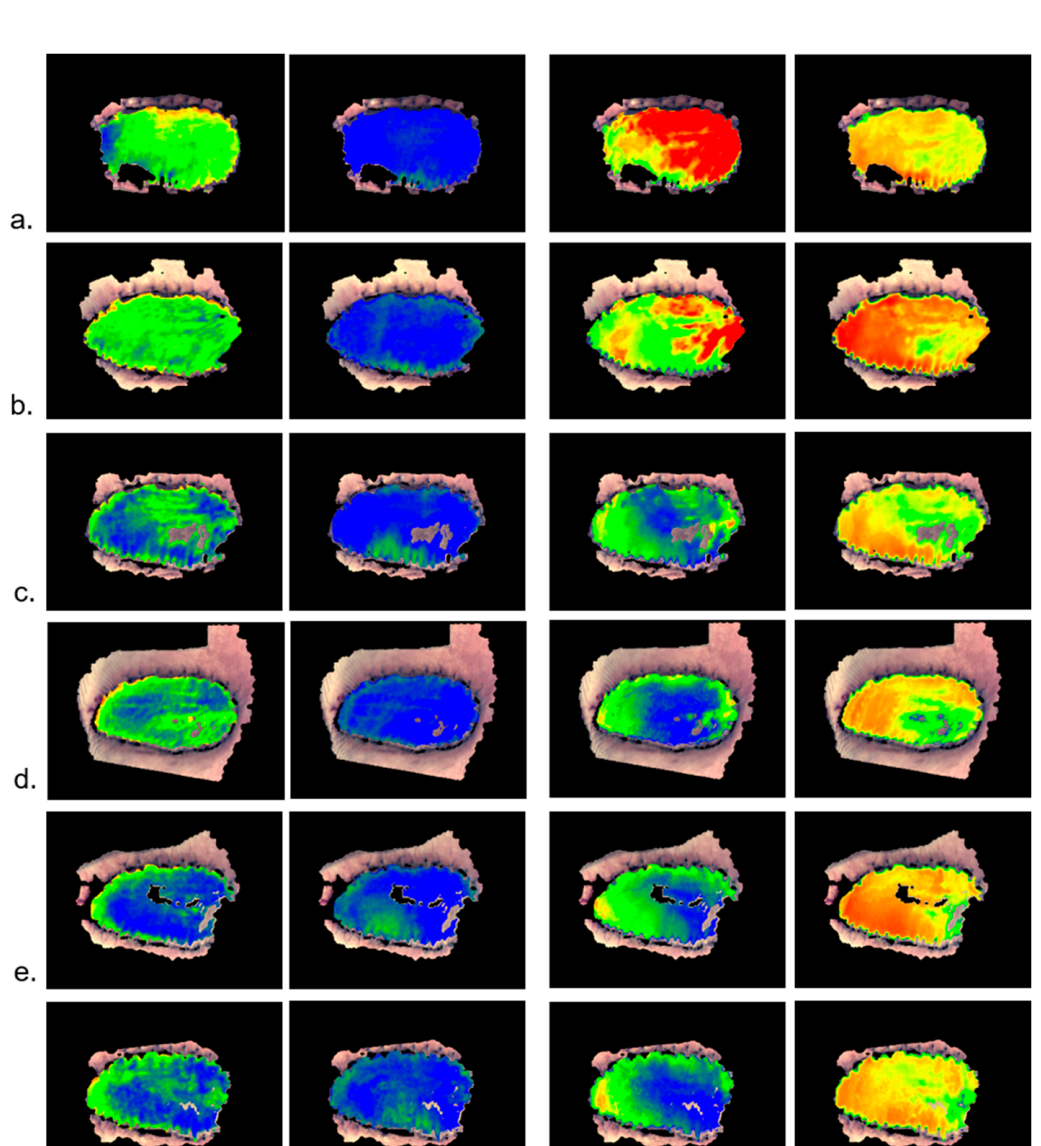
Same transplant measured at the following days (**a**) day 3, (**b**) day 5, (**c**) day 7, (**d**) day 9, (**e**) day 11, (**f**) day 13); vHb_1, xHbO_2__1, vHb_2, xHbO_2__2; color scales for vHb and xHbO_2_, as depicted in Figure 1.

The survey images show the decreasing blood supply from the right side of the flap (Figure 4 vHb_2 and Figure 5a,b vHb_2), clearly indicating a distortion of the arterial conjunction already observable at day 5 (Figure 5b).

The survey images (Figure 5) clearly show the abated arterial blood supply over time. 

The automated analysis is supported by image registration transforming the flap in every image to the same position and dimension. For automated analyses, the complete depth profiles are used.

With this methodology, an advanced procedure for describing and analyzing the perfusion dynamics in flaps is realizable.

#### 2.1.3. Burn Wounds

The extended parameters have been used in a first attempt to generate a classification process for burn wounds. 

Fundamental to the degree of skin damage by heat impact for the healing potential is the remaining perfusion quality in the wound area. With depth profiles, the perfusion situation can be depicted and evaluated on a new, higher level. 

**Figure 6:**
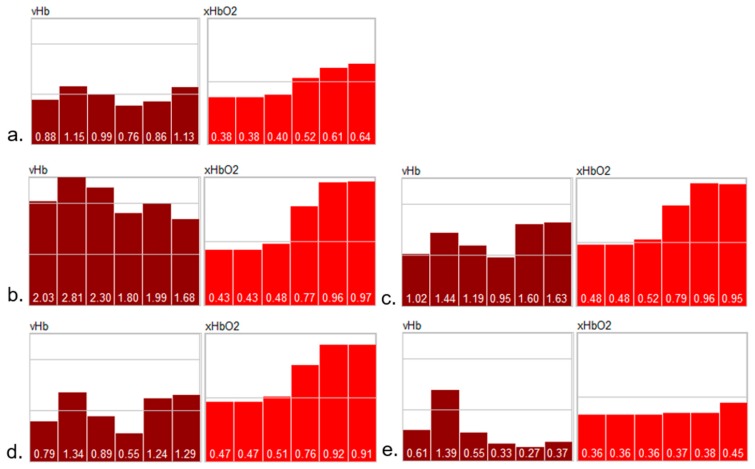
Depth profiles vHb, xHbO_2_ for normal skin (**a**) and different burn degrees (**b**) superficial, (**c**) intermediate (superficial/partial), (**d**) partial-thickness, (**e**) full-thickness).

**Figure 7:**
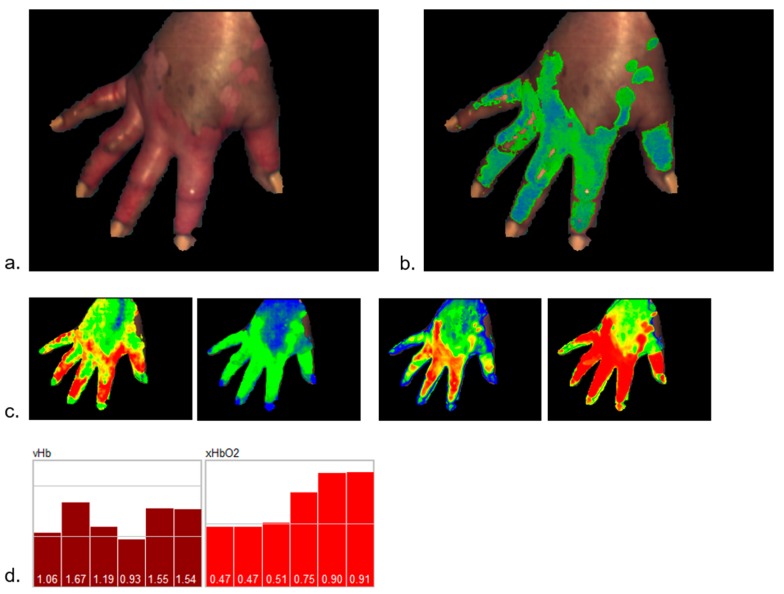
(**a**) Burn wound on a hand, (**c**) perfusion survey images (color scales for vHb and xHbO_2_ as depicted in Figure 1) and (**b**) fuzzy classification (blue: superficial, green: partial-thickness, red: full-thickness); (**d**) perfusion profile from the burn area.

The example shows typical depth profiles of burn wounds with different degrees of damage (burn degrees: superficial, partial-thickness, full-thickness) (see Figure 6), as well as the classification of a burn wound on a hand, clinically assessed to be of partial-thickness (see Figure 7).

This first attempt of a classification process was constructed based on a small number of burn wounds (i.e., approx. 20). Additional to the perfusion parameters, the intrinsic structure parameters of the model were evaluated, and showed characteristic differences between the burn degrees. Also, spectral features, for instance, quantitatively describing the degree of tissue necrosis, are used in the classification process.

The perfusion profiles show significant differences between the burn degrees; for instance, a strong hyperemic reaction for superficial and intermediate partial degrees. With increasing degrees of damage, vHb decreases in all layers; the damage of the deeper vessels is obvious in Figure 6e (full thickness).

Theoretically, with these parameters, an efficient classification can be constructed, but the time after the burn has to be included as a fundamental factor. Especially for intermediate burn degrees, the development of the perfusion in the first 2–3 days is essential for the assessment of the healing potential.

With this methodology, a significant increase in terms of the quantitative and qualitative nature of descriptions and evaluations of burn wounds and wound processes seems to be achievable; therefore, a reliable diagnosis and treatment supporting procedure for burn medicine is foreseeable.

### 2.2. Comparison with Perfusion Parameters Based on a One-Layer Model

Actual standard data processing of hyperspectral imaging spectra involves the calculation of perfusion parameters based on a model consisting of a homogeneous, infinite, one-layer system with hemoglobin as the main component [14,15]. These parameters are comparable, and were validated with other parameters from standard tissue oximetry systems. The perfusion parameters are THI, StO2, and NIR-perfusion. THI (tissue hemoglobin index) denotes the relative volume content of hemoglobin/blood in the measuring volume, StO2 the oxygen saturation of the hemoglobin, and NIR-perfusion a measure of perfusion quality calculated from the NIR-spectral region. The algorithms for THI and StO2 as described in [14] and [15] use wavelength segments from 500 to 800 nm, restricting the depth sensitivity. In the NIR-region (NIR-perfusion), there is no separation of relative volume content and oxygen saturation. 

Because the remission spectra can be completely reproduced by the five-layer model parameters, the THI-, StO2-, and NIR parameters can be principally calculated from the model parameters. THI and StO2 are related to a mixture of the vHb resp. xHbO_2_ of layers 1–4, the NIR-perfusion parameter is a function of (vHb ● xHbO_2_), and vHb and xHbO_2_ of layers 4–5.

Although these one-layer model parameters have been shown to be of high clinical value, the new five-layer model represents a description of the perfusion situation, especially differentiating between the superficial capillary blood volume and oxygen saturation and the parameters of the deeper vessel system. This gives rise to better clinical estimations of perfusion quality, or disturbances of the perfusion system. This additional clinical value will be described for different application areas in subsequent publications.

### 2.3. Wound Healing Disorders

Objective diagnostics in wound healing disorders is a long-term problem with no implications in daily life. The intra- and inter- observer difference is often discussed in the literature [23,24]. A validated, computer-assisted measurement tool based on conventional RGB-imaging has been available for some fifteen years [25,26]. Based on color segmentation, the software is able to quantify the surface, wound borderline, diameter, numeric and percentile part of necrotic tissue, and the fibrin and granulation tissue. Based on this quantification, we are able to analyze the progress of surface reduction, the progression of granulation tissue, and part of the fibrin and necrotic tissue. If the progress of granulation tissue is reduced, we have to check the local therapeutic concept or identify the underlying reasons, e.g., perfusion, edema, oxygenation, infection, etc. 

Additionally, perfusion can be measured by ultrasound and oxygenation with TcPO_2_; however, the examination is time consuming, and edema can only be measured with the circumferences of lower legs.

With hyperspectral imaging, all the following parameters are available within one measurement: perfusion, as described above; based on the characteristic features of the remission spectra, a detailed and quantified segmentation and classification of the wound area, providing portions of necrotic tissue, fibrin, granulation and epithelial tissue; and tissue which is endangered by insufficient perfusion.

The advanced and specific methodology for the clinical application areas addressed in the examples will be described in detail in separate articles.

## 3. Discussion

The requirements for the development of the data processing were:

- The tissue model should describe the physiological structure in a manner which is sufficiently detailed to enable information retrieval, especially concerning the perfusion situation with high clinical value (adequacy);
- The modeling should be able to reproduce real measured remission spectra from skin and wounds over the complete spectrum in detail; the variety of spectra is described in the confidence range, and should sufficiently cover a variety of clinical problems (consistency);
- The solution of the inverse problem should be practicable for imaging measurements with the described measuring geometry in clinical routine environment; the processing should be fast for imaging measurements (practicability).

The challenge is to find a reasonable compromise between the flexibility and adaptivity of the tissue model (many parameters), the physiological informative value, and the physical–mathematical correctness of the solution of the inverse problem.

The described tissue model seems to be sufficiently detailed to offer insights into the perfusion situation, and fulfills the adequacy requirement. The determined model parameters represent perfusion values of the capillary system and the deeper vessel system, and seem to be more informative concerning the perfusion situation.

The values have been proved to be physiologically and clinically plausible (up to now), and the multitude of parameters constitute a better basis for classification processes.

The processing also fulfills the consistency and the practicability requirement. A complete processing of a measurement image with a 50% tissue content needs approx. 10–15 seconds for the 3D physiological perfusion imaging result to be determined.

Many details of the spectrum forms are explainable by the modeling and the dynamics of measuring depth variation over the spectral range. In Λ3, the measuring depth changes very dynamically with the wavelength. Structures such as those at 650nm and 715nm, the rise at 600nm, as well as in Λ_5_ and Λ_6_, are only explainable by the dynamic transition between different layers, and have to be distinguished from biochemical contributions to the spectrum.

The other side of the compromise is that the modeling of the radiation transfer through the system is not physically stringent:

The spectral segments are selected by simple plausibility arguments based on knowledge about the penetration depth in perfused human tissue. By this predetermined dependence upon measuring depth, the spectral segments define the layer thickness relative to the standard value $D_{1}^{0}$.

The heuristic visibility function, enabling the layer separation and differentiation in the successive procedure is based on a theoretical and simulative analysis. The specification of this function, as well as the dependence of the mean path length on the wavelength, is accessible to further refinement and optimization, for instance by expanding to higher orders (taking into account the nonlinearity of the “visibility”). The globally fixed function $D=f_{D}^{A}\left(A\right)$ could be empirically diversified for different spectrum forms and different layer structures.

The use of globalized heuristic functions does not sufficiently correspond to the variety of individual forms of skin systems; the interpretation of the results has to assessed with respect to these limitations.

The parameters have to be empirically validated concerning their physiological interpretation and clinical information content (in consideration of the different clinical context).

Clinically-validated classification processes based on the model parameters will account for gaining confidence in the usability and adequacy of the parameters.

The data processing of HSI remission spectra based on a five-layer model of perfused tissue generates perfusion parameters for every layer and presents them as depth profiles. The evaluation procedure transforms the whole information of the spectrum consistently into model parameters so that the measured spectrum can be completely reproduced.

For the first time, we present a complete system of powerful hyperspectral imaging data acquisition and data processing with high applicability in clinical practice. The main advance of the data processing method is its enhanced information content with highly plausible physiological interpretation and high clinical relevance, which is currently not available with other methods. 

The data processing is integrated into a piece of software running on a computer which is associated with the hyperspectral camera. The data processing requires approx. 10–15 seconds, so that directly after data acquisition, the perfusion parameters are presented to the physician and the patient (bedside diagnostics). 

## 4. Methods and Materials

### 4.1. Hyperspectral Measuring System

All measurements were performed with a HSI-camera TIVITA^®^ Tissue (Diaspective Vision GmbH; Am Salzhaff, Germany) with written consent from volunteers. Data acquisition from patients was conducted in accordance with the Declaration of Helsinki, and the protocol was approved by the Ethics Committee of the Ärztekammer Sachsen-Anhalt, Germany (35/17). All patients gave informed consent. 

The camera was a compact measuring system certified for clinical use [27]. Remission spectra were recorded in the spectral range of 500 to 1000 nm with a resolution of 5 nm; the measuring area was approx. 20 × 30 cm, standard image size was 640 × 480 Pixel, and the recording needed approx. 5 seconds.

### 4.2. Hyperspectral Imaging Data Analysis and Processing

To ensure good qualitative and undisturbed measuring data, the following tests were performed:

- Regular tests of the camera calibration and comparison of spectra from reference objects with corresponding reference spectra.

By software:

- Quality tests of the spectra concerning wavelength-dependent noise to ensure that relevant spectral details for parameter estimation are presented in sufficient quality;
- Tests concerning disturbing influences on the spectra, such as reflection, external light, and strong inclination of parts of the measuring area.

To define adequate quality measures, experimental tests and numerical Monte Carlo simulations [28] were performed.

In the preprocessing procedure of the measuring data, the data quality was tested; data of insufficient quality were excluded from further processing.

#### 4.2.1. Model-Based Analysis

**Figure 8:**
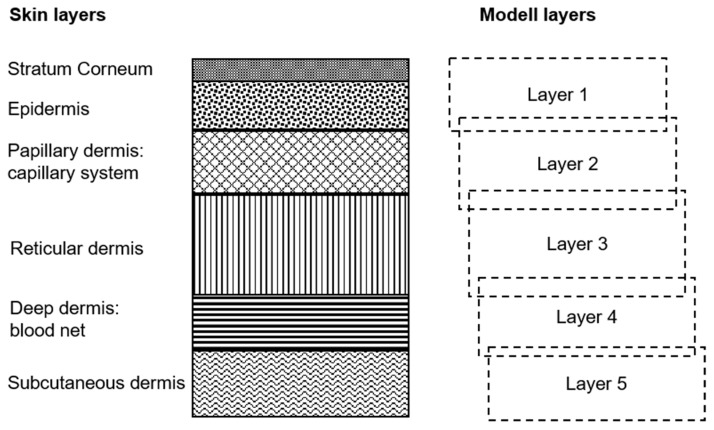
Five-layer skin model.

The skin is modeled as a five-layer-system (Figure 8). Every layer is regarded as homogenous, and is provided with the relevant components:

- Layer 1 (stratum corneum, epidermis): melanin, vHb, and xHbO_2_; vHb denotes the relative volume fraction of total hemoglobin, xHbO_2_ the oxygen saturation of hemoglobin; layer 1 contains also blood and xHbO_2_, because this layer cannot be sufficiently separated from the next;
- Layer 2 (upper dermis: papillary or capillary system): vHb, xHbO_2_, and collagen structure;
- Layer 3 (reticular dermis): vHb, xHbO_2_, and collagen structure;
- Layer 4 (deep dermis, subcutis): vHb, xHbO_2_, vH_2_O, vFat, collagen structure, and connective tissue; vH2O and vFat denote the volume fractions for water and fat;
- Layer 5 (subcutis): vHb, xHbO_2_, vH_2_O, vFat, and connective tissue.

For every layer, the absorption of hemoglobin, water, and fat is explicitly described in a linear approximation; the background absorption and scattering by the collagen matrix, vessels, and connective tissue is jointly described by a linear function containing the so-called intrinsic structure parameters:

(1) $$“\mathrm{Absorbance}”\;A=\ln\left(\frac{R}{I_{0}}\right)=s_{0}+s_{1}l+L\sum_{i}\vartheta_{i}\;\varepsilon_{i}$$

where R: remission, I_0_^:^ incident intensity, $S\left(L\right)=s_{0}+s_{1}\left(L\right)$: intrinsic contributions to absorption and scattering; $\vartheta_{i}$: volume fraction, and $\varepsilon_{i}$ extinction coefficient of component i; L denotes a mean path length, which could be calculated from the path length distribution [29].

Especially for hemoglobin, the derivates Hb ad HbO_2_ are represented in the form

(2) $$\vartheta_{H}\;\left(\varepsilon_{HbO2}+x\;\varepsilon_{Hb}\right)$$

with $\vartheta_{H}$ as the volume fraction of the total haemoglobin and x as the oxygen saturation of the haemoglobin.

The measuring geometry used with this HSI-camera with laminar illumination precludes the separation of different layers by technical control of the path length distribution. The remission at one measuring point is given by an integral over many path length distributions; the measuring volume defined by this distribution varies with the wavelength, depending on the scattering and absorption.

**Figure 9:**
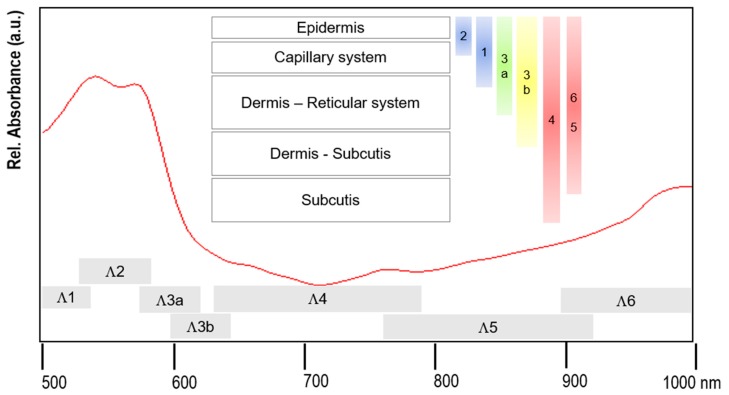
Remission spectrum represented in the “absorbance“ mode. Spectral segments Λ_i_ and schematic: skin layers and approximate measuring depth in the spectral segments.

The form of the spectra is mainly determined by the absorption spectra of hemoglobin (mainly in the range 500–600nm and around 760 nm), as well as by the water and fat absorption spectra increasingly from approx. 700 nm (see Figure 9). The remission spectra contain contributions from the different layers in the measuring volume with a measuring depth depending on the wavelength.

#### 4.2.2. Transformation of the HSI-Remission Spectra

In this modeling, the parameters in L ϑ cannot be determined separately (the scattering is not described explicitly in the model), and depending on the wavelength, L(Λ) may have different values.

Because layer thickness and path length distributions are not explicitly determinable parameters in this model, a depth scale cannot be defined. To obtain a depth profile, we have to make concrete statements about the measuring depth and the path length.

Basis is an analysis of the path length distribution which is dependent on the wavelength to estimate L(Λ); therefore, the measuring depth D is defined as the maximal depth with minimal intensity I_min_: path length distribution h(l,L) Þ L(L)=f(s(L),a(L)); s: scattering; a: absorption; measuring depth D: $\frac{I}{I_{0}}=e^{-a\;L}=I_{min}Þ\;L_{min}=\;\frac{-\ln\left(I_{min}\right)}{a};$ without further knowledge about the dependencies between D and L, the measuring depth D corresponding to the path length L_min_ is supposed to be $D=\;f_{D}^{a}\;L_{min}$.

Because the actual path length distribution is not known, as a first approach, a global function $D\left(L\right)=f_{D}^{A}\left(A\left(L\right)\right)$ (≈ 1a0(L)) is used, including a globally fixed a_0_(λ). Thereby, the measuring depth D for a spectral segment Λ becomes determinable using the total absorbance A of the system (corresponding to the assumption of a homogenous system and the dependence of the measuring depth on μ_a_ (absorption) and μ_s_ (scattering)).

Thereby, different measuring depths can be assigned to different spectral segments:

In the segment 535–585nm (Λ_2_), the measuring depth is least and defines layer 1. The segment 500–535 (Λ_1_) comprises layers 1 and 2, 585–595nm (Λ_3_) layers 1, 2 and 3. The segment 595–690nm (Λ_4_) additionally comprises layer 4. The segment 690–825nm (Λ_5_) comprises all layers (1–5), and segment 825–1000nm (Λ_6_) layers 1–4. 

In Λ_3_, the measuring depth changes very dynamically from layers 3 to 5; therefore, the segment is further subdivided in Λ_3a_ und Λ_3b_.

Due to the higher absorption of H_2_O and fat, the measuring depth is reduced in Λ_5_ und Λ_6_ in comparison to Λ_4_. Water and fat fractions can only be determined in Λ_5_, and especially Λ_6_, with sufficient reliability.

It has to be emphasized once again that the layers are defined by the measuring depths of the spectral segments.

The spectral segments are not strictly fixed, but may be adapted to the actual form of the total absorbance spectrum, and are therefore overlapping.

The different layers contribute differently to the resulting remission spectrum depending on the spectral segments. The nonlinear relation between the layer contributions and the remission spectrum is not explicitly modeled in this framework. Instead, to achieve a separation of the layers, a heuristic function is introduced describing the “visibility” of a lower layer (layer 2) underlying an upper layer (layer 1) in a first order linear approximation:

(3) $$\mathrm{Visibility}\;\mathrm{function}\;f_{V}=f_{v}^{0}{\left(\frac{D_{2}-D_{1}}{D_{1}}\right)}^{\alpha \;}\;{R_{1}}^{\beta }e^{-a_{1}L_{1}}$$

where D_1_ denotes the measuring depth (layer thickness) of layer 1 and D_2_ of layers 1 and 2, L_1_ is the mean path length corresponding to the measuring depth D_1_, and R_1_ the remission of layer 1.

In a first approximation, the pathways through layer 2 are only affected in layer 1 by absorption a_1_, and therefore, by path length L_1_ (β = 0). The exponent α, determining the volume portion of layer 2 relative to layer 1, is globally fixed. 

The determination of the parameter of the visibility function is based on comparison with Monte Carlo simulations of two-layer systems.

Because D_1_ resp. L_1_ are not known for individual measurements, standard values are defined: L10=D10fDA.

In the first step, for every spectral segment, a numerical adaptation to a homogenous equivalent system (i.e., a homogeneous, one-layer model with the relevant components) is performed. The adaptation quality is a measure of the appropriateness of the segment selection, and therefore, for the layer structure. Inside the segment, the dependence on the measuring depth should be low. 

For the approximate determination of the layer contributions, two-layer modeling is performed successively for the underlying layers:

1. The volume captured by Λ_2_ is defined as layer 1. From the remission R_1_(Λ_2_), the parameters $S_{1}\left(L_{2}\right)\;and\;\vartheta_{1}\;L_{1}\left(L_{2}\right)$ $(L_{1}\left(L_{2}\right)=L_{1}^{0}$) are determined.
2. In Λ_1_, layer 2 is also captured; the combined remission R_12_(Λ_1_) can be presented in the form $R_{12}\left(L_{1}\right)=R_{1}\left(L_{1}\right)+f_{V}\left(L_{1}\right)\cdot R_{2}\left(L_{1}\right)$, with $f_{V}$ as the visibility function. R_1_(Λ_1_) results from $S_{1}\left(L_{1}\right)\;and\;\vartheta_{1}\;L_{1}\left(L_{1}\right)$, with $L_{1}\left(L_{1}\right)=f_{L}\left(L\right)\;L_{1}^{0}$.

From the remission spectrum $R_{2}\left(L_{1}\right)=\frac{R_{12}\left(L_{1}\right)-R_{1}\left(L_{1}\right)}{f_{V}\left(L_{1}\right)}$, the parameters of layer 2 are determined. The parameters refer to the path length $\Delta L_{2}=L_{2}\left(L_{1}\right)-L_{1}\left(L_{1}\right)$ (approximately), and are finally scaled with respect to the standard value $L_{1}^{0}$. a_1_ L_1_ in the visibility function is determined using the total absorbance A(Λ_1_).

3. In the further segments, i.e., Λ_3_ etc., the further layers (3, etc.) are successively captured. The processing is analogue to 2. ($R_{123}=R_{12}+f_{V}\left(L_{3}\right)\;R_{3},\;etc.$).

From this result, the component parameters of the layers are (s_0_, s_1_, {$\vartheta_{i}\;L_{1}^{0}\;\}$) and the visibility function $f_{V}\left(L_{i}\right)$ (parameter D) for the actual spectral segments is Λ_i_. D is the mean measuring depth of the actual layer. A depth range [D_min_…D_max_] for every layer is stored. With these values and the global function $f_{D}^{A}\left(A\right)$, the spectral segments can be reconstructed, with A as the absorbance of the total HES.

#### 4.2.3. Reconstruction of the Spectrum

With the spectral segments, the layer parameters and the visibility function, the complete spectrum can be reconstructed successively:

1. With Λ_2_ and the parameters of layer 1, R_1_(Λ_2_) is calculated.
2. With Λ_1,_ the parameters of layer 2, D_2_(Λ_1_), R_2_(Λ_1_), and A_1_(Λ_1_), R12(Λ_1_) is calculated.

Based on R12, the parameters of HES12(Λ_1_) are determined.

3. With Λ_3_ and the parameters of layer 3, R_3_(Λ_3_) is calculated from D_2_(Λ_3_) and D_3_(Λ_3_), as well as R12(Λ_3_) and A12(Λ_3_). With HES12, R123(Λ_3_) is calculated.

Analogue processing for the further layers.

**Figure 10:**
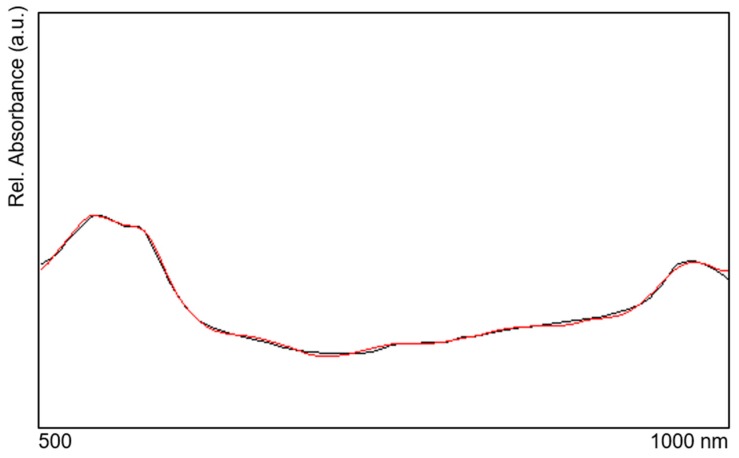
Measured spectrum (absorbance) (red) and reproduced spectrum by the model (black).

**Consistency:** With the model parameters determined by this process, the measured spectrum can be reproduced nearly perfectly (see Figure 10). This means that the information contained in the spectrum is practically completely transformed into the model parameters. 

**Uniqueness:** Generally, there is no unique adaptation maximum in the (model) parameter space, especially for the spectral segments, except Λ_2_ and partially Λ_5_ and Λ_6_. To reduce potential ambiguity, the pathway between the actual maximum and the successively following maximum for the next spectral segment is estimated by additional intermediate segments (not fulfilling the requirement of quasi-stationarity with respect to the measuring depth). Thereby, the actual valid maximum can be selected with a higher level of probability.

Principally, in each case, even the global maximum of adaptation cannot be regarded as the “true“ solution due to the limited reality of the modeling system. 

#### 4.2.4. Parameters and Confidence Range of Modeling

The parameters vHb und xHbO_2_ named in the layer model are related to the model parameters: vHb = $\vartheta_{H1}\;L_{1}^{0}$; xHbO_2_ ≡ x in formula (2).

Because $L_{1}^{0}$ is a globally fixed parameter, vHb represents an index value (range [0...2.5]); the x-values are in the range [0...1].

The physiologically-acceptable variation ranges of the model parameters define the variety of spectrum forms representable by the model. In the processing procedure, every real spectrum is proved to be within this confidence range before further processing. 

The reproduction quality of the spectrum is a test of consistency.

## 5. Conclusions

Despite the aforementioned limitations, the presented processing method provides a more differentiated outcome in relation to the perfusion situation in the layered tissue structure, and comprehensively utilizes the information content of hyperspectral measuring data.

The examples show the potential for creating a new, valuable, clinical procedural and investigative category in different medical fields. The processing is a further step for establishing hyperspectral imaging in medicine, and considers the measuring conditions and essential requirements for clinical practicability.

To create a supporting powerful diagnosing system using hyperspectral imaging technology, model-based data processing has to be complemented by an efficient, knowledge-based method.

## 6. Further Validations and Developments

A fundamental problem is the lack of reliable and accurate reference methods for detailed perfusion values in the layers. A systematic comparison with a Monte-Carlo simulation is in progress, as well as a comparison with spectroscopic measurement methods, enabling control of the measuring depth, and other methods depicting the layer structure, e.g., OCT.

Methodical progressions concern the improvement of the modeling of radiation transport, the separation of the layers, and more generally, a reduction of the described limitations.
